# Comparative Proteomic Identification of Mature and Immature Sperm in the Catfish *Cranoglanis bouderius*

**DOI:** 10.1371/journal.pone.0151254

**Published:** 2016-03-10

**Authors:** Jintao Chen, Aiguo Zhou, Shaolin Xie, Chao Wang, Zijun Lv, Jixing Zou

**Affiliations:** 1 College of Animal Science, South China Agricultural University, Guangzhou, Guangdong, China; 2 Qingyuan North River Fishery Science Institute, Qingyuan, Guangdong, China; CAS, CHINA

## Abstract

To understand the molecular responses of mature and immature sperm in the catfish *Cranoglanis bouderius*, we used the iTRAQ proteomics approach to perform proteomic profiling of spermatogenesis in *C*. *bouderius*. As a result, 1,941 proteins were identified, including 361 differentially expressed proteins, 157 upregulated proteins and 204 downregulated proteins in mature sperm relative to immature sperm. All of the identified proteins were categorized into seven types of subcellular localizations and three molecular functions and were found to be involved in nine biological processes. All of the differential proteins were involved in 235 different pathways. Moreover, we found that the tricarboxylic acid (TCA) pathway played an important role in the energy metabolism of sperm and that the EABB pathway was involved in the mechanism of spermatogenesis. Our study is the first to use the iTRAQ-based proteomic approach to analyze the catfish sperm proteome, and the results we obtained using this approach are valuable for understanding the molecular mechanisms of fish spermatogenesis.

## Introduction

Teleost spermatozoa play an indispensable role in gametogenesis and fertilization. Spermatozoa are a type of polarized and specified cell. Unlike somatic cells, spermatozoa have only a minimal amount of cytosol and lack organelles that contain the endoplasmic reticulum, Golgi apparatus, lysosomes, peroxisomes and ribosomes [1, 2]. This structure makes spermatozoa a preferable model for thremmatology research. The fundamental biomolecules of spermatozoon include different proteins that are intricately related to many biological functions such as integrality, fertilization and spermatozoon mobility. More recent proteomics research has used mass spectrometry to analyze and characterize the sperm proteome [3–5]. However, thus far, sperm proteome analyses have not been widely reported.

The motility and fertility of mature spermatozoa originate during spermatogenesis. During the period of spermatogenesis, immature spermatids turn into mature spermatozoa through a series of external and intrinsic changes in structure. The most obvious morphological characteristic is the appearance of a flagellum that can be used to effectively distinguish oval spermatids from mature spermatozoa. Despite the important role of spermatogenesis in the spermatozoa fertilization of teleost fish, the species-specific cellular, molecular and biochemical mechanisms remain unknown [6, 7].

*C*. *bouderius* is a rare cultured catfish from south China, and its captive breeding is incipient. The potential commercial value of this species warrants further research on its reproduction. Spermatogenesis and spermioteleosis play key roles in reproduction, and the differences between mature and immature sperm might provide useful information for understanding these processes. In the present study, a proteomics approach was used to perform proteomic profiling of the spermatogenesis of *C*. *bouderius*. We investigated the mature and immature sperm proteins using a non-gel-based quantitative proteomics technique termed isobaric tags for relative and absolute quantification (iTRAQ). This approach can be used to precisely qualify and quantify different proteins and to explore potential protein interactions [8]. This is the first time that the iTRAQ-based proteomics approach has been used for the catfish sperm proteome. This information is valuable for understanding the molecular mechanisms of fish spermatogenesis.

## Materials and Methods

This study was approved by the animal ethics committee of South China Agricultural University.

### Sample preparation

The experimental catfish (*C*. *bouderius*) were obtained from the fish hatchery of the Qingyuan North River Fishery Science Institute, Qingyuan, Guangdong, China. The fish were maintained in the recirculation system of the College of Animal Science, South China Agricultural University, under standard laboratory conditions until they were sampled. Tricaine methanesulfonate (MS 222) is a common anaesthetic that we used for catfish zoopery in accordance with the Bureau of Sport Fisheries and Wildlife [9]. Six live *C*. *bouderius* from 2 different testis developmental stages were euthanized with an overdose of MS 222 at 300 mg/L (buffered with sodium bicarbonate) for sampling. Testes were obtained by dissection. Sperm suspensions were prepared from homogenized testes with phosphate buffered saline. The presence or absence of flagella was used to confirm the morphologies of the mature or immature sperm, respectively, using light microscopy. The suspension was centrifuged at 1,500 x g for 15 min at 4°C to collect the centrifuged deposit. A millilitre of lysis buffer containing 8-M urea and 1x Protease Inhibitor Cocktail (Roche Ltd. Basel, Switzerland) was added to the samples, followed by sonication on ice and centrifugation at 13,000 rpm for 10 min at 4°C. The supernatant was transferred to a fresh tube. All of the procedures and animal handling were performed in accordance with the Guide for the Chinese Association for Laboratory Animal Sciences. Approval of the study was obtained from the Animal Ethics Committee of South China Agricultural University.

### Protein digestion and TMT labelling

The protein concentrations of the supernatants were measured using a BCA protein assay, and then 100 μg of protein per condition was transferred to a new tube and adjusted to a final volume of 100 μL with 100 mM TEAB (triethyl ammonium bicarbonate). After the addition of 5 μL of 200 mM TCEP (trichloroethyl phosphate), the samples were incubated at 55°C for 1 hour, and then 5 μL of 375-mM iodoacetamide was added to the samples for incubation for 30 min in the dark at room temperature. For each sample, proteins were precipitated using ice-cold acetone and then were redissolved in 100 μL of TEAB. Next, the proteins were digested with sequence-grade modified trypsin (Promega, Madison, WI), and the resulting peptide mixture was labelled using chemicals from the TMT Reagent Kit (Pierce Biotechnology, Rockford, USA). Proteins were labelled with TMT as follows: 14_1, 14_2, 14_3, NC_1, NC_2, and NC_3, and each was labelled with 126 to 131 isobaric tags. The labelled samples were combined and dried *in vacuo*.

### High pH reverse phase separation

The peptide mixture was redissolved in buffer A (20-mM ammonium formate in water, pH 10.0, adjusted with ammonium hydroxide) and then fractionated using high pH separation with the Aquity UPLC system (Waters Corporation, Milford, MA) connected to a reverse phase column (XBridge C18 column, 2.1 mm x 150 mm, 3.5 μm, 300 Å, Waters Corporation, Milford, MA). The high pH separation was performed using a linear gradient that started from 5% buffer B to 35% buffer B over 40 min (buffer B contained 20 mM ammonium formate in 90% ACN, pH 10.0, adjusted with ammonium hydroxide). The column was re-equilibrated at initial conditions for 15 min. The column flow rate was maintained at 200 μL/min, and column temperature was maintained at room temperature. Fifteen fractions were collected, and each fraction was dried in a vacuum concentrator for the next step [10].

### Low pH nano-HPLC-MS/MS analysis

The fractions were resuspended with 40 μL of solvent C (solvent C contained water with 0.1% formic acid; D contained ACN with 0.1% formic acid), separated using nanoLC and analysed using online electrospray tandem mass spectrometry. The experiments were performed on a Nano Aquity UPLC system (Waters Corporation, Milford, MA) connected to a quadrupole Orbitrap mass spectrometer (Q-Exactive) (Thermo Fisher Scientific, Bremen, Germany) equipped with an online nano-electrospray ion source. A 10-μL peptide sample was loaded onto the trap column (Thermo Scientific Acclaim PepMap C18, 100 μm x 2 cm) with a flow rate of 10 μL/min for 3 min and subsequently separated on an analytical column (Acclaim PepMap C18, 75 μm x 15 cm) with a linear gradient from 2% D to 40% D over 165 min. The column was re-equilibrated at initial conditions for 15 min. The column flow rate was maintained at 300 nL/min, and the column temperature was maintained at 40°C. An electrospray voltage of 2.2 kV versus the inlet of the mass spectrometer was used. The Q-Exactive mass spectrometer was operated in the data-dependent mode to switch automatically between MS and MS/MS acquisition. Survey full-scan MS spectra (m/z 350–1200) were acquired with a mass resolution of 70K, followed by 15 sequential high energy collisional dissociation (HCD) MS/MS scans with a resolution of 17.5K. In all cases, one microscan was recorded using a dynamic exclusion of 30 s. MS/MS fixed first mass was set at 100.

### Database searching

Tandem mass spectra were extracted using MM_File_Conversion.exe version 3.9 build 25. Charge state deconvolution and deisotoping were not performed. All MS/MS samples were analysed using Mascot (Matrix Science, London, UK; version 2.3.0). Mascot was set up to search the *Siluriformes* database (version 2014-5-28, 17,443 entries) assuming digestion with the trypsin enzyme. Mascot was searched with a fragment ion mass tolerance of 0.050 Da and a parent ion tolerance of 10.0 PPM. Carb amidomethyl of cysteine and TMT 6 plex of lysine and the n-terminus were specified in Mascot as fixed modifications. Oxidation of methionine was specified in Mascot as a variable modification.

### Criteria for protein identification

Scaffold (version Scaffold_4.3.4, Proteome Software Inc., Portland, OR) was used to validate MS/MS based on the peptide and protein. Peptide identifications were accepted if they could be established at greater than 47.0% probability to achieve a false discovery rate (FDR) less than 1.0% using the Scaffold Local FDR algorithm. Protein identifications were accepted if they could be established at greater than 99.0% probability and contained at least 2 identified peptides. Protein probabilities were assigned by the ProteinProphet Algorithm [11]. Proteins that contained similar peptides and that could not be differentiated based on MS/MS analysis alone were grouped to satisfy the principles of parsimony. Proteins sharing significant peptide evidence were grouped into clusters. For peptide identification quality control, the percolator algorithm was used to the maintain peptide FDR levels at lower than 1%. Only unique peptides were used for protein quantification, and normalization on the protein median was used to correct for experimental bias. The minimum number of proteins that must be observed was set to 100. Low scoring spectra and peptides without labelling were eliminated by filtering based on a protein FDR ≤ 0.01 and a peptide FDR ≤ 0.01 [12]. Because iTRAQ quantification might have underestimated the amount of real fold change between the labelled samples [13], a protein with a difference > 1.5-fold and with a p-value < 0.05 was regarded as being differentially expressed in our analysis.

### Data Analysis

The annotation of GO and KEGG information for each protein was derived from the UniProtKB/Swiss-Prot database, and then an enrichment analysis of GO and KEGG pathway annotations was conducted using our in-house software based on a hypergeometric test [14]. Gene ontology (GO) categories were assigned to the identified proteins based on the TAIR GO slim provided by blast2GO. KEGG pathway analysis was performed using KASS (KEGG Automatic Annotation Server http://www.genome.jp/tools/kaas/).

## Results

### Differential protein expression profile with gene ontology annotation

**Fig 1:**
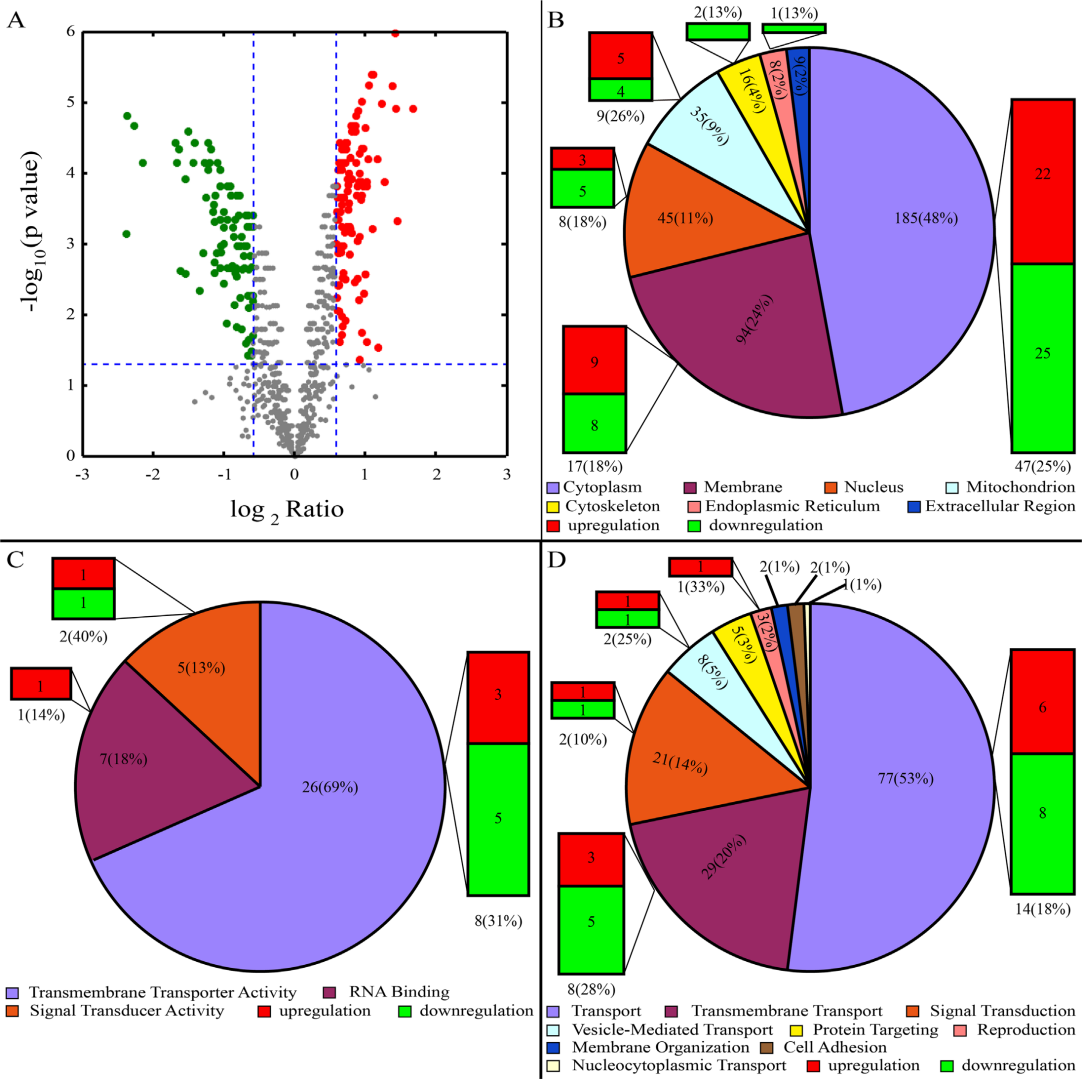
GO analysis of differential protein profile. (A) Volcano Plot of differential protein profile, The logarithmic ratios of the protein intensities were plotted against the negative logarithmic p-values of the t-test performed from triplicate experiments. Blue dotted lines separate specific differential proteins marked as red (upregulated) and/or green (downregulated) dots from the background (grey dots). (B) Predicted subcellular localization of proteins. (C) Predicted molecular functions classification of proteins. (D) Predicted biological process classification of proteins. Digit in pie chart indicated the quantity of total proteins and their percentage. Histogram showed the quantity of differential proteins and their percentage, red meant upregulation, downregulation in green.

We performed TMT protein labelling and MS to identify a potential quantitative proteomic approach for profiling the proteins of mature and immature sperm samples. A total of 2,369 proteins were obtained, and 1,941 annotated proteins were found in both the mature sperm and immature sperm, which matched to 1,792 gene groups. After filtering, 361 unique proteins were identified as differentially expressed between the mature and immature sperm (S1 Appendix). Among all of the differentially expressed proteins, 157 proteins in the mature group were upregulated and 204 proteins in the mature group were downregulated relative to the immature group. A volcano plot represents the results of the differentially expressed proteins. The logarithmic ratios of the protein intensities were plotted against the negative logarithmic p-values of the t-test performed from triplicate experiments. Blue dotted lines separate specific differential proteins marked as red (upregulated) and/or green (downregulated) dots from the background (grey dots). The upregulated and downregulated proteins demonstrated significant ratios in combination with high reproducibility (positive log2 ratios) (Fig 1A).

All proteins were classified using GO slim for cellular localization, molecular functions and biological processes.

The subcellular localization analysis revealed that 185 of the proteins were located in the cytoplasm (48% of the total proteins), which included 47 differentially expressed proteins (56% cytoplasmic proteins), and 22 differentially expressed proteins were upregulated while 25 were downregulated. A total of 92 proteins were located at the membrane (24%), which included 17 differentially expressed proteins (20%), of which 9 proteins were upregulated and 8 proteins were downregulated. A total of 45 proteins were located at the nucleus (11%), which included 8 differentially expressed proteins (18%), of which 3 were upregulated and 5 were downregulated. A total of 35 proteins were located in the mitochondria (9%), which included 9 differentially expressed proteins (26%), of which 5 proteins were upregulated and 4 proteins were downregulated. A total of 16 of proteins were located in the cytoskeleton (4%), which included 2 downregulated differential proteins (13%). A total of 8 proteins were located at the endoplasmic reticulum (2%), which included only a single downregulated differentially expressed protein (13%). A total of 9 proteins were located at the extracellular region (2%), but none of the differentially expressed proteins were involved (Fig 1B).

Molecular function classification grouped the proteins into 3 different main families, of which 69% had transmembrane transporter activity, 18% were related to RNA binding and 13% were involved in signal transduction activity. There were 8 differentially expressed proteins with transmembrane transporter activity, of which 3 were upregulated and 5 were downregulated, 1 was an upregulated differentially expressed protein with RNA binding activity and 2 were differentially expressed proteins with signal transduction activity, of which 1 gene was upregulated and the other was downregulated (Fig 1C).

The biological process category comprised 77 proteins that were enriched for transport activity (53% of total proteins), which included 14 differentially expressed proteins (18% of the transport category), of which 6 were upregulated and 8 were downregulated. A total of 29 proteins were enriched for transmembrane transport activity (20%), which included 8 differentially expressed proteins (28%), where 3 were upregulated and 5 were downregulated. A total of 21 proteins were enriched for signal transduction activity (14%), which included 2 differentially expressed proteins (10%), of which 2 were upregulated and 1 was downregulated. A total of 8 proteins were enriched for vesicle-mediated transport activity (5%), which included 2 differentially expressed proteins (25%), of which 1 was upregulated and the other was downregulated. A total of 5 proteins were enriched for protein targeting activity (3%), and 3 proteins were enriched for reproductive functions (2%), which included 1 upregulated protein. Proteins without differential expression were enriched for membrane organization activity, cell adhesion and nucleocytoplasmic transport activities, which included 2 (1%), 2 (1%) and 1 (1%) proteins, respectively (Fig 1D).

### Differential protein expression profile with KEGG analysis

**Fig 2:**
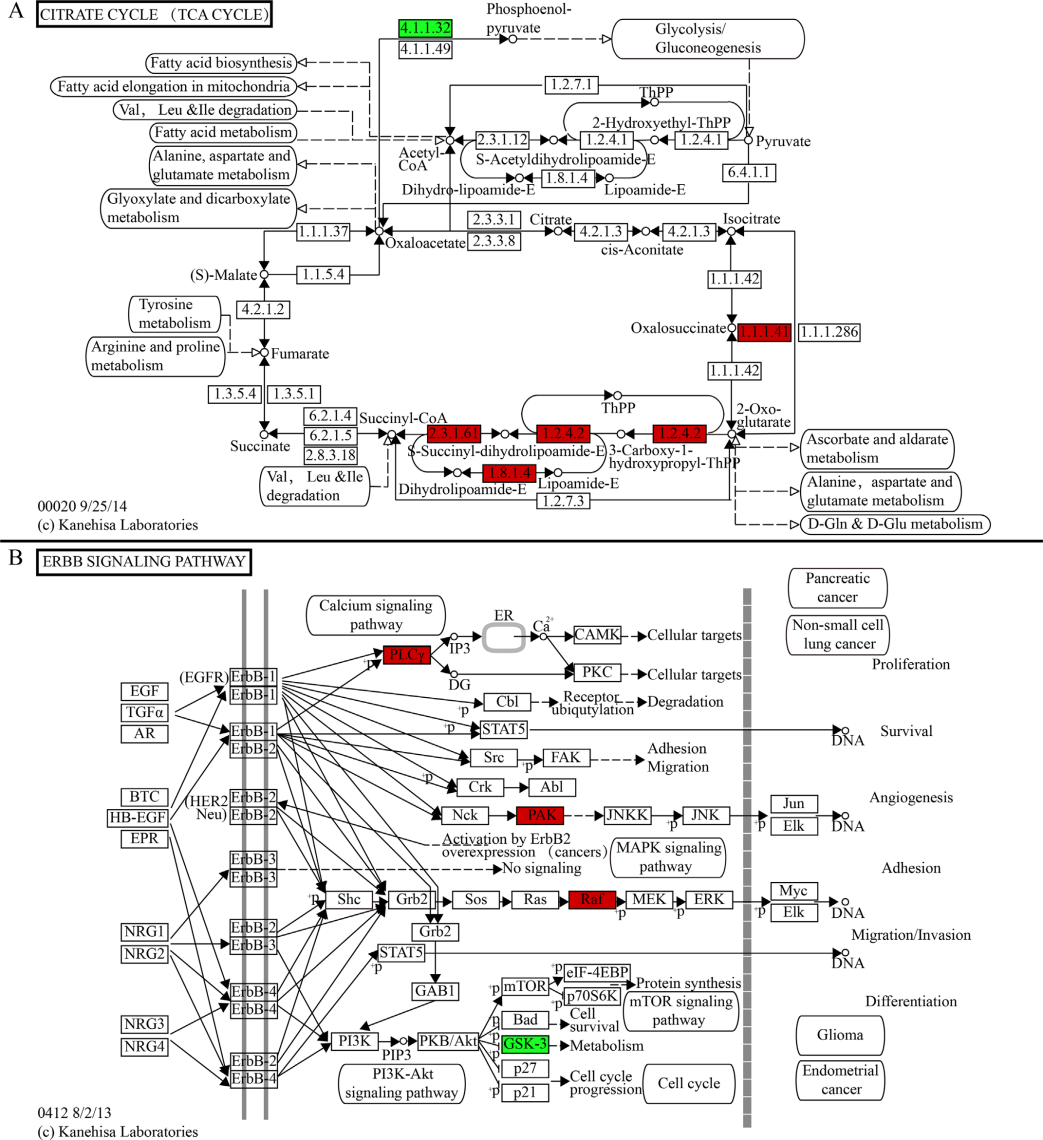
KEGG analysis of differential proteins. (A) Differential proteins involved in the oxidative phosphorylation pathway, 4.1.1.32: phosphoenolpyruvate carboxykinase (GTP), 1.1.1.41: isocitrate dehydrogenase (NAD+), 1.2.4.2: 2-oxoglutarate dehydrogenase E1 component, 2.3.1.61: 2-oxoglutarate dehydrogenase E2 component (dihydrolipoamide succinyltransferase), 1.8.1.4: dihydrolipoamide dehydrogenase. (B) Differential proteins involved in the ERBB pathway. Block in red meant upregulation, downregulation in green.

To further characterize the potential functions of the sperm proteins, the *C*. *bouderius* sperm proteome was analysed for KEGG pathways. Within the proteome, 235 pathways were assigned. Analysis of the pathways relative to the TCA cycle identified 5 differentially expressed proteins that were crucial for sperm metabolism. Moreover, PLCγ, PAK, Raf and GSK-3 of the ERBB pathway were essential for spermatogenesis and maturity (Fig 2A and 2B).

## Discussion

Spermatogenesis and spermioteleosis are complex and precisely regulated processes that involve numerous changes in cellular morphology and cellular components. A large variety of proteins are involved in this metabolic process. Because of its specific simple structure, the spermatozoa became an ideal model to investigate the proteins that are involved in spermatogenesis and spermioteleosis. Proteomics technologies offer an extraordinary opportunity to investigate the molecular mechanisms that regulate sperm function. In the present study, we investigated protein variety and abundance in mature and immature spermatozoa and explored the major proteins that participate in spermatogenesis and spermioteleosis.

In the current study, a TMT-labelling quantitative proteome analysis was performed on mature and immature spermatozoa. As a result, 1,941 proteins were identified, including 361 differentially expressed proteins. All of the identified proteins were categorized into 7 subcellular localizations, 3 molecular functions and 9 biological processes. Differentially expressed proteins were distributed in the cytoplasm, membrane, nucleus, mitochondria, cytoskeleton and endoplasmic reticulum. The proteins were involved in transmembrane transporter, RNA binding and signal transduction activities, in addition to, vesicle-mediated transport, cell adhesion and nucleocytoplasmic transport activities. The differentially expressed proteins were involved in 235 different pathways. Because of the abundance of information, manual analysis of each protein and pathway was not feasible, and thus analyses that identify specific crucial proteins and functions in spermatozoa should be performed to assess their contribution to reproduction.

Energy consumption is required for the full metabolic processes of spermatogenesis and spermioteleosis. The TCA cycle is one of the crucial pathways for energy supply [15]. Our study identified 3 different proteins that were involved in the TCA cycle, including isocitrate dehydrogenase (IDH, ec: 1.1.1.41), α-ketoglutarate dehydrogenase complex (α-KGDHC) and phosphoenolpyruvate carboxykinase (PEPCK, ec: 4.1.1.32). IDH and α-KGDHC were upregulated, and PEPCK was downregulated.

IDH and α-KGDHC are rate-limiting enzymes in the TCA cycle [16, 17]. NAD+ or NADP+-dependent IDH catalyses isocitrate to ketoglutarate (2-oxoglutarate) through oxidative decarboxylation, which is accompanied by the generation of CO_2_ and NADH or NADPH [18]. Production of ketoglutarate cannot occur without IDH [19]. The α-KGDHC complex is composed of 3 subunits, namely, ketoglutarate dehydrogenase (E1), which is also designated as oxoglutarate dehydrogenase (OGDH; EC 1.2.4.2) [20], dihydrolipoamide acyltransferase (E2, DLST; ec: 2.3.1.61) and dihydrolipoamide dehydrogenase (E3, DLD; ec: 1.8.1.4) [21]. The α-KGDHC enzyme is highly regulated and determines metabolic flux through the TCA cycle. α-KGDHC catalyses the conversion of α-ketoglutarate to succinyl-CoA, which is accompanied by the production of NADH, a process that directly provides electrons for the respiratory chain [22]. As a result of IDH and α-KGDHC activity, isocitrate is oxidatively decarboxylated to ketoglutarate and, soon afterwards, decarboxylated to succinyl-CoA, which participates in the TCA cycle [23]. The abundance of IDH and α-KGDHC in organs has a direct influence on the efficiency of oxygen oxidation and energy metabolism. Upregulation of IDH and α-KGDHC is indicative of a higher rate of oxygen oxidation and energy metabolism, which would enhance the physiological ability of sperm.

PEPCK is the rate-limiting enzyme in gluconeogenesis [21]. The metabolism of pyruvate and/or lactate is necessary for the capacitation of mammalian spermatozoa, and glucose can decrease the capacitation reaction [24]. Spermatozoa prefer to use pyruvate or lactate over glucose. When glucose is sufficient, most of it appears to be metabolized [25]. In our study, the lower concentration of PCK1 may be related to the capacitation of spermatozoa; consequently, spermatozoa prefer to metabolize glucose rather than synthesis glucose. As a result, more pyruvate and/or lactate can provide sufficient energy to supply zymolyte for capacitation.

Based on the obtained data, we found that the expression of PLCγ, PAK, Raf and GSK-3 observably changed in the ERBB signalling pathway. PLCγ, PAK and Raf were upregulated, while GSK-3 was downregulated. The expression of each gene is involved in a crucial signalling pathway, and these 4 signalling pathways were closely related to spermatogenesis and spermioteleosis.

A calcium-dependent isotype of PLCγ is activated during sperm capacitation [26]. The activation of PLC leads to the hydrolysis of phosphatidylinositol 4,5 -bisphosphate (PIP2) into diacylglycerol (DG) and inositol triphosphate (IP3) [27], which elevates Ca^2+^ levels. The formation of IP3 and the elevation of Ca^2+^ levels might reinforce PLC activation through the mobilization of an acrosomal Ca^2+^ pool. At the same time, the formation of DG activates specific PKC isoforms [28, 29]. As a result, a Ca^2+^ channel is activated on the sperm plasma membrane [30]. PKC increases the intracellular pH by accelerating the Na+/H+ exchange [31]. The alkalization of sperm that is mediated by Na+/H+ exchange is responsible for flagellar beating [32]. PKC regulation of flagellar motility was demonstrated in sea urchin and human sperm [33, 34]. The mechanism of spermatozoon-triggered Ca^2+^ increase as a conduit for Ca^2+^ entry into the egg cytoplasm was supported by frog and fish studies, in which sperm-derived PLC induced the generation of a Ca^2+^ wave [35]. Based on our analysis, the regulation of PLCγ might promote sperm motility.

P21-activated kinases (PAKs) belong to a highly conserved serine/threonine kinase family that is crucial for various signalling processes [36]. One pathway that is related to the maintenance of sperm viability is the c-Jun NH2-terminal kinase (JNK, also known as SAPK) pathway. In this pathway, PAK can phosphorylate the JNK activators (MKK4, also referred to as SEK1/JNKK). Biochemical studies demonstrated that the phosphorylation of MKK4 activates JNK [37]. The activation of JNK phosphorylates the NH_2_-terminal activation domain of c-Jun [38] on Ser63 and Ser73, which causes the increase of c-Jun transcriptional activity [39]. During the life of spermatozoa, the osmolality of the external environment usually changes dramatically. As a result, sperm probably developed a cellular defence mechanism against osmotic stress that includes the JNK pathway [40]. PAK also activates the mitogen-activated protein kinase (MAPK) cascades by affecting the actin and tubulin cytoskeletons, which leads to transcription and cytoskeletal structure changes [41]. The dysfunction of the MAPK pathway can cause spermatogenetic damage [42]. As the upstream regulator of the JNK and MAPK pathways, PAK is essential for sperm survival and quality. Mature spermatozoa are fully differentiated cells that lack active transcriptional machinery. Hence, upregulation of PAK that leads to JNK and MAPK cascades might be an important posttranscriptional mechanism in sperm.

Extracellular signal-regulated kinases (ERKs) belong to a family of mitogen-activated protein kinases (MAPKs). These kinases are widely present in spermatozoa and are important for flagellar motility, hyperactivation and the acrosome reaction of mature spermatozoa [43]. The ERK pathway includes 3 pivotal kinases, namely Raf (as MAPK kinase kinase), MEK (as MAPK kinase), and ERK1 and 2 (as MAPK) [44]. Raf can be activated through a direct interaction with phosphatidic acid or can be independent from an association with Ras [45]. The specific phosphorylation mechanisms of MAPK cascades that are initiated by RAF have been widely reported. Raf phosphorylates the 218 and 222 serine residues of MEK [46], and the latter phosphorylation leads to the activation of ERK through extreme high-specificity phosphorylation [47]. The mitotic proliferation of primary spermatogonia and the acquisition of sperm motility during spermatogenesis, which were attributable to the expression and activation of ERK1/2, could be observed in mice [48]. On the other hand, ERK1/2 in fowl spermatozoa regulates flagellar motility by phosphorylating axonemal and/or accessory cytoskeletal proteins [49]. Moreover, the ERK1/2 pathway possibly exerts an effect on the tyrosine phosphorylation of fibrous sheath proteins during capacitation [50]. Therefore, we deduced that the upregulation of Raf would activate the ERK pathway and ultimately influence sperm motility and capacitation.

Glycogen synthase kinase 3 (GSK3) is related to sperm motility and is present in sperm and testis in several species [51]. GSK3 exerts a severe inhibitory effect on flagellar movements by adjusting the phosphorylation levels of glucose metabolism enzymes. For example, inhibition of GSK3 activity through phosphorylation leads to a significant increase in the percentage of rapid- and medium-speed spermatozoa, as well as all sperm velocity parameters and coefficients [52]. GSK3 is a critical downstream element of the PI3K/AKT1 cell survival pathway. PI3K activates a downstream kinase, which, together with PKB/Akt, leads to the deactivation of GSK3 and might contribute to capacitation [53, 54]. GSK3 also activates a mammal-specific isoform of type1 protein phosphatase (PP1γ2). PP1γ2 is specifically expressed in sperm and testis, and it is an indispensable enzyme in the acquisition and regulation of sperm motility [55]. Mouse sperm with GSK3 knocked out demonstrates an elevation in the catalytic activity of PP1γ2 [56]. As a result, the downregulation of GSK3 that was observed in our study may correspond to an elevation in sperm motility during capacitation.

Increasing evidence shows that spermatozoa are a potentially useful model to investigate reproductive control in general. Proteomics represents an extraordinary tool for evaluating the molecular mechanisms regulating sperm function. The development of contraceptive vaccines [57] and diagnoses of sperm dysfunction, asthenozoosperm or fertilization failure using proteomics have been carried out in clinical trials [58–60]. In the present study, we compared the differentially expressed proteins between the mature and immature sperm of *C*. *bouderius*. We analysed the subcellular localization, molecular function and biological processes of differentially expressed proteins and deduced the potential functions of crucial proteins in specific signalling pathways. Protein data derived from proteomic approaches are valuable for evaluating the exact mechanisms of the specific proteins that control fertility. Western blotting or qPCR may be better for revealing the expression levels of these important proteins, as the results of our study suggest that iTRAQ may not be optimal. However, the data we obtained is sufficient to indicate that some key differentially expressed proteins could affect spermatogenesis and spermioteleosis. The identified proteins should be strictly evaluated in subsequent studies to verify their specific functions in spermatozoa, was well as their role in reproduction. Moreover, confounding factors that can affect proteomics data should also be considered when translating the discoveries from proteomic analyses into clinical trial research and practice. Taken together, the results of the present study may provide new avenues for the management of male reproduction in general and novel targets for expected reproductive outcomes.

## Supporting Information

S1 Appendix Data of VolcanoPlot. (XLSX)
